# Patient satisfaction with a clinically integrated sleep apnea care model vs. the current sleep care paradigm

**DOI:** 10.3389/frsle.2024.1534441

**Published:** 2025-01-22

**Authors:** Gregory D. Salinas, Wendy Cerenzia, Brandon Coleman, Frances Thorndike, Samantha Edington, Heidi Riney

**Affiliations:** ^1^CE Outcomes, LLC, Birmingham, AL, United States; ^2^Nox Health, Inc., Alpharetta, GA, United States

**Keywords:** survey, sleep apnea, patient, comprehensive care, continuous positive airway pressure (CPAP), satisfaction

## Abstract

**Introduction:**

Sleep apnea can have severe negative health effects, including cardiovascular diseases, metabolic disorders, and decreased quality of life. Continuous positive airway pressure (CPAP) therapy is highly effective and the gold standard treatment for sleep apnea; however, traditionally fragmented sleep healthcare has resulted in low levels of treatment adoption and adherence. A recent white paper analysis of traditional health plan claims found that a comprehensive model significantly outperformed traditional health plans with higher rates of adoption (80 vs. 49%), adherence (62 vs. 25%), and persistence (53 vs. 11%) to CPAP therapy, which resulted in lower total healthcare costs. To understand the patient experience in these models of care, this study compared patient satisfaction between the traditional sleep care approach and a clinically integrated, comprehensive sleep care program.

**Methods:**

A survey was developed to understand differences in the patient experience with the two different care models with respect to: access to sleep care, including time from initial appointment to seeing a sleep specialist, referral and insurance process; ease of sleep testing process and receiving a diagnosis; adoption, quality of education, and training with CPAP; ongoing adherence support with CPAP, and quality of life. Data were compared using descriptive statistics and Chi-square analyses.

**Results:**

A significantly higher proportion of patients in the comprehensive model were satisfied with all measured points in the patient's journey. Notably, twice as many patients in the comprehensive model were very satisfied with: ease of navigating the testing process, time between diagnosis and CPAP adoption, insurance navigation for CPAP approval, and availability and level of ongoing CPAP support. Comprehensive care patients experienced fewer work disruptions due to sleep apnea: only 7% missed work in the past 3 months, compared to 58% in the traditional model.

**Discussion:**

Overall, the study highlights the benefits of a comprehensive care model in improving patient satisfaction with their sleep apnea journey and overall quality of life for individuals with sleep apnea. Pairing this positive patient experience data with prior data from the same treatment model shows that removing obstacles within a patient's journey positively impacts satisfaction while simultaneously improving adherence rates and reducing total healthcare costs.

## Introduction

Adequate sleep is vital for sustaining overall health and wellbeing (Ramar et al., 2021), significantly influencing cognitive performance (Alhola and Polo-Kantola, 2007; Lowe et al., 2017), emotional stability (Vandekerckhove and Wang, 2017), and physical health (Clement-Carbonell et al., 2021). Disruptions to sleep, such as sleep apnea, a highly prevalent condition characterized by repeated interruptions in breathing during sleep, can have severe negative health effects. Untreated sleep apnea is associated with an increased risk of cardiovascular (hypertension, heart disease, stroke), metabolic (diabetes), pulmonary, and neuropsychiatric disorders (Guo et al., 2013; Gottlieb et al., 2010; Yeghiazarians et al., 2021; Bonsignore et al., 2019; Stone et al., 2016; Shan et al., 2015; Tan et al., 2018; Brennan et al., 2022; Chang et al., 2013; Liguori et al., 2021; Gleeson and McNicholas, 2022; McNicholas, 2019). Sleep apnea can also lead to daytime fatigue, impaired cognitive function, and decreased quality of life. Addressing sleep apnea through appropriate diagnosis and treatment is vital to mitigate these health risks and improve overall wellbeing.

Despite a high prevalence and effective treatment, the picture is complicated by a significant undiagnosed and untreated population. Though the impact of sleep apnea on negative health outcomes is well-known, the current healthcare system often falls short in effectively identifying patients. A study put forth by the American Academy of Sleep Medicine (AASM) indicated 80% of people go undiagnosed (American Academy of Sleep Medicine, 2016).

Traditional sleep care management is fragmented, fraught with time delays, and uncoordinated; the patient is often left unsupported (Ye et al., 2022). These systemic problems have been attributed to several barriers to effective sleep apnea management, including limited access to sleep physicians and diagnostic services (particularly in remote areas), key members of the care experience working in different departments and thus lacking care coordination, socioeconomics, lack of awareness about the condition, lengthy time delays in the process of getting to a diagnosis and on treatment, lack of CPAP device training to help patients adopt treatment, patient perception of efficacy, and lack of ongoing support to help patients achieve optimal adherence (Weaver and Grunstein, 2008; Palm et al., 2021; Billings et al., 2011; Engleman and Wild, 2003; Mehrtash et al., 2019). These challenges prevent many patients from achieving long-term success in managing their sleep apnea, which has negative impacts on overall health due to apnea's frequent comorbidity with other chronic health conditions (Bonsignore et al., 2019). These events, in turn, drive up total healthcare costs and utilization leading to an annual sleep apnea economic burden of $149 billion in the United States alone (American Academy of Sleep Medicine, 2016).

Continuous positive airway pressure (CPAP) therapy is the gold standard treatment and is highly effective in treating sleep apnea (Patil et al., 2019); however, the traditionally fragmented approach to sleep healthcare has led to low rates of treatment adoption and adherence, hindering improvements in clinical outcomes. A recent claims study compared rates of adoption and adherence to CPAP among those diagnosed with sleep apnea in the traditional sleep care paradigm vs. those in a clinically integrated sleep care model (Risk Strategies Consulting, 2024). The results showed that only 49% adopted PAP therapy in the traditional sleep care model vs. 80% in the clinically integrated care model; only 25% remained on therapy in the traditional model after the 1st year vs. 62% in the integrated care model; and only 11% continued on therapy by the end of year 2 in the traditional sleep care model vs. 53% in the integrated care model. They also showed that patients that were adherent to CPAP therapy were able to achieve lower total healthcare utilization in the first two years of therapy to $2,743 per patient per year on an age, gender, and risk-adjusted basis.

It is probable that the fragmentation barriers patients encounter along their journey may result in frustration and dissatisfaction with their experience, resulting in poor treatment adherence. Possible solutions to this problem include alternative care models, such as a comprehensive, clinically integrated care model, that eliminate these barriers and improve treatment adoption, adherence, and patient satisfaction.

This survey study aims to explore the factors behind these challenges and their impact on patients by comparing patient satisfaction in two different sleep care models: the traditional sleep care paradigm (patient is responsible for navigating insurance, scheduling appointments for each provider, working with an external durable medical equipment (DME) provider to obtain their CPAP device and necessary supplies, and adopting/troubleshooting this equipment) vs. a patient-centric, clinically integrated comprehensive sleep care environment. By analyzing patient satisfaction through each step of the journey, from diagnosis of sleep apnea to ongoing treatment, the study seeks to identify the specific barriers that hinder successful adoption and adherence to CPAP therapy. This comparison between the traditional care model and the comprehensive clinically integrated approach aims to reveal insights into how a more comprehensive, patient-centered system may improve patient satisfaction with access to care, ease of testing/diagnosis, treatment adoption/quality, ongoing support, and quality of life.

## Methods

### Description of integrated sleep care model

The comprehensive care model involves a clinically integrated network of specialists under one umbrella, which includes board-certified sleep physicians to diagnose and treat patients, a medical management team led by a medical director and nurse practitioner to handle medical exceptions/barriers to therapy, respiratory therapists, and a behavioral care team with oversight by a behavioral sleep specialist, to coordinate a multi-step process including evaluation, telehealth consultation with a sleep physician, home sleep testing, personalized treatment device fulfillment, device education, training, troubleshooting as needed, and ongoing support provided to the patient by the care team (Risk Strategies Consulting, 2024). For the home sleep testing, electroencephalography (EEG) and electrooculography (EOG) are used to ensure a conclusive Apnea-Hypopnea Index (AHI) for those patients that have low pre-test probability of having sleep apnea or have inconclusive sleep testing with a traditional home sleep test. CPAP device data is reviewed to ensure that the sleep apnea is being well-treated and patients can be switched to advanced CPAP devices or check pulse oximetry on PAP therapy if needed.

### Survey design and development

A survey was developed to provide insight into the patient's experience and satisfaction with key points in the patient journey from access to a sleep physician, sleep disorder testing and diagnosis, CPAP delivery and training to ease treatment adoption, ongoing support to troubleshoot issues and promote adherence, and quality of life. Question types included multiple-choice, Likert scale, and free text response. Respondents were asked to supplement their responses with written information for certain questions and to provide overall comments at the end of the survey. The protocol for this study was determined to be exempt from review by WCG/WIRB (Puyallup, WA) under 45 CFR § 46.104(d)(2).

### Survey distribution and data collection

To achieve the sample from the comprehensive care model, users were obtained from a proprietary database of patients engaged in the Nox SleepCharge program by Nox Health. To achieve the sample of the traditional care model, survey respondents were recruited from June through August 2024 via targeted ads on social media channels. Patients were eligible for the study if they were between the ages of 21–65, US residents, able to read and speak English, had private insurance through an employer, and were diagnosed with sleep apnea by a clinician (self-reported). The patient age range and necessity for private insurance was to eliminate potential confounders of government-based insurance (Medicare/Medicaid) to more evenly compare to the private insurance-based comprehensive model.

The survey was expected to take ~10 min to complete. A monetary incentive (equivalent to US $15) was offered to respondents for their participation.

### Data analysis

Descriptive statistics were conducted on key items of the survey, using Chi-square analyses for categorical variables and *t-*tests for continuous variables. Analyses were conducted to compare responses between the comprehensive care model and the traditional model. Statistical analysis was conducted using Qualtrics (Provo, UT). Values were considered significant when *P* < 0.05. Open-ended comments were coded by theme by the research team and pulled out to help explain descriptive results.

## Results

### Demographics of survey respondents

A total of 206 patients with sleep apnea were included in the analysis of this study: 102 in the traditional sleep apnea care model and 104 in the comprehensive model. The demographics of patient respondents (Table 1) are similar in regards to age (mean years of 44 in the traditional care group vs. 48 in the comprehensive care group), years with sleep apnea (mean years of 5 for both), gender, and race/ethnicity. The groups only differed with respect to marital status: 76% of the respondents in the comprehensive care model were married, compared to only 43% of those in the traditional model.

**Table 1:** Sample demographics.

	**Traditional care sample (*n* = 102)**	**Comprehensive model sample (*n* = 104)**
Age in years (mean)	44	48
Years with sleep apnea (mean)	5	5
**Gender**		
Female	49%	49%
Male	51%	51%
**Race/ethnicity (select all that apply)**		
American Indian	1%	2%
Asian/Asian American	5%	6%
Black/African American	17%	12%
Hispanic/Latino/Spanish origin	11%	6%
White	71%	79%
Other/prefer not to say	1%	3%
**Marital status**		
Single (never married)	29%	14%
Married	43%	76%
Divorced	21%	8%
Widowed	6%	2%
**Use of CPAP**		
Never used a CPAP device	14%	7%
Used CPAP in the past but not currently	32%	3%
Currently use CPAP	54%	90%

While the primary study inclusion was a diagnosis of sleep apnea, there was no requirement related to CPAP use. The study found, however, that self-reported CPAP usage differed between the two groups: 90% of those in the comprehensive model were current users of CPAP compared to 54% of those in the traditional model. Nearly a third of those in the traditional model had used CPAP in the past but did not use it at the time of the survey.

### Satisfaction with access to care

Respondents were asked to comment on their satisfaction with multiple points in the patient journey (Table 2). Those in the comprehensive care model were significantly more likely to be satisfied with the time from initial appointment to the appointment with a sleep specialist, the referral process/communication with the sleep medicine specialist, insurance related to the sleep test, ease of the sleep testing process, and the time from the sleep test to receiving a sleep apnea diagnosis (*P* < 0.05).

**Table 2:** Satisfaction with initial care, access, and sleep testing process.

	**Traditional care sample (*n* = 102)**	**Comprehensive model sample (*n* = 104)**
**Access to sleep testing**		
**Time from initial appointment to getting appointment with sleep specialist** ^*^		
Not at all satisfied	7.8%	1.0%
Somewhat/moderately satisfied	53.9%	14.5%
Very/extremely satisfied	38.2%	84.5%
**Referral process and communication with your sleep medicine specialist** ^*^		
Not at all satisfied	9.8%	1.9%
Somewhat/moderately satisfied	50.0%	14.6%
Very/extremely satisfied	40.2%	83.5%
**Insurance authorization/approval of the sleep test** ^*^		
Not at all satisfied	10.8%	0.0%
Somewhat/moderately satisfied	29.4%	5.8%
Very/extremely satisfied	59.8%	94.2%
**Time from getting a prescription for a sleep test to having the sleep test** ^*^		
Not at all satisfied	8.8%	1.0%
Somewhat/moderately satisfied	29.4%	13.5%
Very/extremely satisfied	59.8%	85.6%
**I am satisfied with my current access to care for my sleep apnea** ^*^		
Strongly disagree/disagree	30.4%	4.9%
Neutral	18.6%	9.7%
Agree/strongly agree	51.0%	85.4%
**The sleep testing process**		
**Time it took from getting a sleep test to receive a diagnosis of sleep apnea** ^*^		
Not at all satisfied	3.9%	0.0%
Somewhat/moderately satisfied	47.1%	8.7%
Very/extremely satisfied	49.0%	91.3%
**Ease of the sleep testing process** ^*^		
Not at all satisfied	11.8%	1.9%
Somewhat/moderately satisfied	50.0%	15.4%
Very/extremely satisfied	38.2%	82.7%

^*^P < 0.05.

Eighty-five percent of the patients in the comprehensive model, compared to 51% of those in the traditional model, agreed or strongly agreed that they were satisfied with their access to care (*P* < 0.05).

Some patients in the traditional group wrote comments indicating a frustration with accessing a sleep specialist:

- “Everything requires a referral, Too many loopholes to go through.”
- “Getting an appointment is nearly impossible unless you're willing to wait months. I have had to research and troubleshoot any problems on my own.”

### Satisfaction with the sleep testing process and receiving a diagnosis

Less than half of the traditional sample (49%) had a high level of satisfaction with the time it took to get a sleep specialist appointment, compared to 91% of the comprehensive sample (*P* < 0.05). Only 38% of the traditional sample indicated high satisfaction with the ease of the sleep testing process, compared to 83% of the comprehensive care sample (*P* < 0.05; Table 2). Patients in the traditional group indicated difficulties with the testing process, including insurance coverage:

- “I had one sleep test that was invalid due to a poor setup because of a tech and my sleep doctor would not redo the test, even though the results were incorrect because of that poor setup.”
- “My health provider did not verify that the home sleep test was covered by my insurance, now I have to pay out of pocket. When I finally got the appointment, I found out that I have to go for another sleep study in order to get a CPAP and it could be months.”
- “The whole process took too long, the doctor said my insurance would cover my CPAP machine I ordered… but it never covered it no matter how the claim was submitted.”

The patients in the comprehensive group sometimes commented on the uncomfortable nature of the test itself but did not mention any difficulties with testing or treatment access:

- “Setting up the test at home would need some help from a relative or friend. Would have been nice to get a heads up before getting scheduled/before signing up for at home test so we can factor in the decision.”
- “The test itself was uncomfortable due to having things attached to you. Otherwise, the process was easy.”

However, the patients in the comprehensive study had many positive comments about the process as well:

- “What an amazing quick process, working with friendly “live” people helping all the way.”
- “Quick and simple. Was guided through entire process.”
- “Having private work sponsored programs help a lot in access and easier follow up.”

### Satisfaction with CPAP adoption and quality of care

A subset of patients who had experience with CPAP were asked to rate their satisfaction with multiple components of the process of receiving and using CPAP, including time from diagnosis to receiving a CPAP machine, insurance authorization of the device/vendor, care/communication of the CPAP trainer and respiratory therapist, and quality of education provided to them about sleep apnea. Similar to the diagnostic process, the patients in the comprehensive care model were significantly more satisfied than the traditional patient sample with their experience receiving CPAP (*P* < 0.05; Table 3).

**Table 3:** Satisfaction with experience receiving CPAP.

	**Traditional care sample (*n* = 88)**	**Comprehensive model sample (*n* = 97)**
**CPAP adoption**		
**Time from diagnosis of sleep apnea to receiving a CPAP machine** ^*^		
Not at all satisfied	6.8%	0.0%
Somewhat/moderately satisfied	50.0%	8.2%
Very/extremely satisfied	43.2%	91.8%
**Insurance authorization/approval of the CPAP machine or CPAP vendor** ^*^		
Not at all satisfied	8.0%	1.0%
Somewhat/moderately satisfied	46.6%	6.2%
Very/extremely satisfied	45.5%	92.8%
**The care and communication of your CPAP trainer and respiratory therapist** ^*^		
Not at all satisfied	14.8%	1.0%
Somewhat/moderately satisfied	42.0%	21.6%
Very/extremely satisfied	43.2%	77.3%
**The quality of the education provided to me related to sleep apnea and expectations of a CPAP machine** ^*^		
Not at all satisfied	12.5%	4.1%
Somewhat/moderately satisfied	48.9%	27.8%
Very/extremely satisfied	38.6%	68.0%
**I am satisfied with my current quality of sleep apnea care received from my provider** ^*^^†^		
Strongly disagree/disagree	33.3%	3.9%
Neutral	22.5%	11.7%
Agree/strongly agree	44.1%	84.5%
**Ongoing support to ensure adherence**		
**Troubleshooting challenges when starting CPAP therapy (fit of mask, amount of air)** ^*^		
Not at all satisfied	12.5%	4.1%
Somewhat/moderately satisfied	48.9%	27.8%
Very/extremely satisfied	38.6%	68.0%
**Availability and level of support received in continuing use of CPAP therapy** ^*^		
Not at all satisfied	17.0%	2.1%
Somewhat/moderately satisfied	43.2%	18.6%
Very/extremely satisfied	39.8%	79.4%

^*^P < 0.05.

^†^Asked to complete sample of respondents (n = 102 traditional patients; n = 104 comprehensive model).

The entire sample of respondents was asked about their perception of the current quality of care received from their provider. A significantly higher proportion of those in the comprehensive sample (85%) compared to 44% of those in the traditional sample indicated that they agree/strongly agree that they are satisfied with their care (*P* < 0.05).

### Satisfaction with ongoing support

Sixty-eight percent of patients in the comprehensive model had high levels of satisfaction with troubleshooting challenges when starting CPAP therapy, significantly higher than the 39% of those in the traditional model who were satisfied in this area (*P* < 0.05). Furthermore, there was a large gap between these groups in satisfaction around the availability and level of support received in the continued use of CPAP therapy (79 vs. 39%, *P* < 0.05).

Some comments from those in the traditional group highlight why they may be dissatisfied with the quality of their ongoing support:

- “I feel like I have nobody that takes my concerns seriously. I cannot use the [CPAP] machine so I don't know what other route to take to handle my sleep apnea. I feel lost and do not know where to turn for help. My quality of life is not good.”
- “I never really felt like my provider cared to keep up with my diagnosis. The communication on their end was bad and they never followed up with me after I mentioned that I could not use the CPAP machine.”
- “They won't speak to you on the phone. You always have to come in. They don't seem to monitor your device data, unless you come in.”

### Satisfaction with quality of home and work life

Respondents were asked their agreement with several statements around satisfaction with their overall quality of life and the level to which sleep apnea disrupts their ability to work and family life. Patients in the comprehensive care model cohort were significantly less likely to agree that sleep apnea disrupts their work/family life (*P* < 0.05, Table 4).

**Table 4:** Quality of home and work life.

	**Traditional care sample (*n* = 102)**	**Comprehensive model sample (*n* = 104)**
**I am satisfied with my quality of life** ^*^		
Strongly disagree/disagree	34.3%	12.5%
Neutral	24.5%	19.2%
Agree/strongly agree	41.2%	68.3%
**Sleep apnea disrupts my family life** ^*^		
Strongly disagree/disagree	25.5%	41.7%
Neutral	14.7%	16.5%
Agree/strongly agree	59.8%	41.7%
**Sleep apnea disrupts my ability to work** ^*†^		
Strongly disagree/disagree	24.5%	47.5%
Neutral	23.5%	17.8%
Agree/strongly agree	52.0%	34.7%
**I have missed one or more days of work in the past 3 months because of sleep apnea** ^*†^	58%	7%

^*^P < 0.05.

^†^Only asked of respondents who are currently working (n = 87 traditional patients; n = 93 comprehensive model).

The respondents who were currently employed were asked whether sleep apnea has caused them to miss work. Over half of patients in the traditional sample indicated that sleep apnea has caused them to miss at least 1 day of work in the past month, compared with only 7% of patients in the comprehensive care sample (*P* < 0.05).

### Role of CPAP use on satisfaction

In order to understand if satisfaction was correlated solely with the patients being on CPAP therapy and no other aspects of the comprehensive care model, respondents were divided into 3 different groups: traditional model patients currently not on CPAP (*n* = 47), traditional model patients currently using CPAP (*n* = 55) and patients in the comprehensive care model currently using CPAP (*n* = 94; Table 5). When comparing these subgroups, numerically the trend for levels of satisfaction increases from the lowest rates in the traditional with no CPAP cohort, the middle rates of satisfaction in the traditional with CPAP cohort, and the highest rates of satisfaction observed among those in the comprehensive model using CPAP. For example, 28% of those in the traditional group not on CPAP agreed/strongly agreed that they were satisfied with their quality of life; 52% of patients in the traditional group on CPAP agreed/strongly agreed; and 70% of patients in the comprehensive care model agreed/strongly agreed. 34% of those in the traditional group not on CPAP agreed/strongly agreed that they were satisfied with their current access to sleep apnea care, compared to 66% of patients in the traditional group on CPAP and 87% of patients in the comprehensive care model. Regarding satisfaction with the current quality of their sleep apnea care, 26% of those in the traditional group not on CPAP agreed/strongly agreed that they are satisfied, compared to 60% of patients in the traditional group on CPAP and 86% of patients in the comprehensive care model.

**Table 5:** Role of CPAP on satisfaction with care.

	**Traditional care sample not on CPAP (*n* = 47)**	**Traditional care sample on CPAP (*n* = 55)**	**Comprehensive model sample (*n* = 94)**
**I am satisfied with my quality of life**			
Strongly disagree/disagree	40.4%	29.1%	11.7%
Neutral	31.9%	18.2%	18.1%
Agree/strongly agree	27.7%	52.7%	70.2%
**I am satisfied with my current access to care for my sleep apnea**			
Strongly disagree/disagree	44.7%	18.2%	3.2%
Neutral	21.3%	16.4%	9.6%
Agree/strongly agree	34.0%	65.5%	87.2%
**I am satisfied with my current quality of sleep apnea care received from my provider**			
Strongly disagree/disagree	48.9%	20.0%	2.2%
Neutral	25.5%	20.0%	11.7%
Agree/strongly agree	25.5%	60.0%	86.2%
**Sleep apnea disrupts my family life**			
Strongly disagree/disagree	28.8%	21.8%	44.7%
Neutral	17.0%	12.7%	14.9%
Agree/strongly agree	53.2%	65.5%	40.4%
**Sleep apnea disrupts my ability to work**			
Strongly disagree/disagree	19.1%	29.1%	47.5%
Neutral	27.7%	20.0%	17.8%
Agree/strongly agree	53.2%	50.9%	34.7%

Further, 53% of those in the traditional group not on CPAP agreed/strongly agreed that sleep apnea disrupts their ability to work, 51% of patients in the traditional group on CPAP agreed/strongly agreed, and 35% of patients in the comprehensive care model agreed/strongly agreed. Fifty-three percent of those in the traditional group not on CPAP agreed/strongly agreed that sleep apnea disrupts their family life, 66% of patients in the traditional group on CPAP agreed/strongly agreed, and 40% of patients in the comprehensive care model agreed/strongly agreed. In summary, being on CPAP outside of the comprehensive care model does not automatically indicate satisfaction with sleep apnea care, although the cohort is more satisfied than those not receiving CPAP treatment.

## Discussion

Overall, patients receiving treatment in the clinically integrated comprehensive care model were significantly more likely than patients in a traditional care model to be satisfied with all aspects of sleep apnea treatment, including: [a] access to care, [b] ease of the sleep testing process, [c] CPAP adoption, training, and ongoing treatment, [d] ongoing support to ensure adherence to CPAP therapy, and [e] quality of home and work life. Further, twice as many patients in the clinically integrated care model were very or extremely satisfied with the ease of navigating the testing process, the amount of time between diagnosis and receipt of a CPAP machine, navigation of authorizations and approvals for the CPAP machine, and the availability and level of support received during ongoing CPAP treatment compared to those in a traditional sleep apnea care approach. To our knowledge, this is the first study comparing patients' perceptions of two different models of sleep care, providing insight into how clinically integrated and coordinated care is experienced by the patient, including their self-reported experiences with CPAP therapy adoption and ongoing treatment.

While there was a significant difference between care groups in the proportion of patients currently on CPAP treatment, this may not be surprising given the aim of the clinically integrated model to overcome barriers and help patients adopt and adhere to treatment. In a *post-hoc* exploratory analysis that removed traditional care patients who were not using CPAP, the significant difference in satisfaction remained, with patients in the comprehensive care model reporting higher satisfaction than those in the traditional model and on CPAP.

Patients sampled from the comprehensive care model reported being more satisfied with their quality of life than their counterparts in the traditional model. Patients in the comprehensive care model also reported that sleep apnea had less of a negative impact on work. Whereas, half of the patients in the traditional model reported that sleep apnea disrupted their ability to work, roughly a third of those in the comprehensive model indicated that impairment. Further, only 7% of those in the comprehensive model said they missed at least 1 day of work in the past 3 months due to sleep apnea, compared to 58% of the traditional patient sample. This fits with research over the past decade highlighting how sleep disorders, particularly untreated sleep disorders, can negatively impact employee safety, productivity, and absenteeism (Silva et al., 2021). Employers are beginning to see the value in successfully treating employees with sleep disorders (Berger et al., 2012; Garbarino et al., 2016; Kales and Czeisler, 2016).

The two samples used in this comparison were similar in their age, gender, race/ethnicity, and length of time with OSA. A major difference between the two groups is current marital status: patients in the comprehensive care sample were much more likely to be married than those in the traditional sample. While no direct causality has been shown between sleep apnea and divorce, multiple studies have identified and examined this relationship. An online survey conducted by AASM found that 35% of adults in the United States sleep in another room to accommodate a bed partner; 33% go to sleep at an earlier or later time than desired (American Academy of Sleep Medicine, 2023). A Portuguese study has suggested that initiation of sleep apnea treatment prompted couples to return to a shared bedroom (Cascais Costa et al., 2023). A recent study presented at the 2024 SLEEP conference suggested that adherence to PAP treatment is linked to higher satisfaction in a relationship (Troxel et al., 2024). Thus, one could infer higher satisfaction rates with the sleep apnea process could lead to higher adoption and retention of sleep apnea therapy, improving patient relationships with their partners.

Pairing this high satisfaction data with research from another study with the same comprehensive care model yields a more complete picture of how a coordinated care model can overcome barriers. According to a study of traditional health plan claims, adults diagnosed with apnea achieved low levels of adoption [49%], adherence [25%], and persistence (2 or more years of adherence) to apnea therapy [11%] (Risk Strategies Consulting, 2024). In contrast, patients in the same comprehensive care model described in this study demonstrated higher rates of adoption [80%], adherence, [62%], and 2 or more years of adherence (persistence) to therapy [53%] (Risk Strategies Consulting, 2024). Importantly, when patients adopt, adhere, and persist in CPAP therapy, total healthcare utilization declines, which is reflected in lower healthcare costs. The claims study described above found that, on an age-, gender-, and risk-adjusted basis, the difference in total healthcare costs for members with apnea who adhere to therapy compared to those who are non-adherent is significant: $2,743 per patient per year (Risk Strategies Consulting, 2024).

Limitations of this study include introducing selection bias through the use of targeted ads on social media channels to recruit patients for the traditional care cohort. While attempts were made to obtain comparable demographics between the two cohorts, the study was intentionally not a randomized controlled trial as it was intended to assess real-world attitudes and satisfaction in two different care models. Future studies could directly involve sleep apnea clinics to target those in a traditional model. Limiting the respondents to those with private insurance was to better compare the two models, but could limit the generalizability of the results to a 65+ age group that commonly experiences sleep apnea. Further, the analysis relies on self-reported data, which could be subject to recall bias and inaccuracies, but that is the nature of patient satisfaction data. Despite these potential limitations, the recruitment of the sample confirms that sleep apnea affects a diverse demographic of people. It should also be noted that the comprehensive model uses a home sleep study. While well-monitored and perhaps more efficient than a clinic-based study, a home-based model may be less discriminating for those with more severe cases of sleep apnea or multiple comorbidities.

In conclusion, a sleep apnea program that coordinates and simplifies the patient experience, accelerates the time from appointment to adherence, and removes the barriers to patient success leads to increased patient satisfaction with the diagnosis process (eg, time between diagnosis and doctor/sleep study appointments), ease of process (no need for referral for each step and prior authorization/insurance navigation), and communication with a sleep specialist team (including education/treatment troubleshooting). While there continues to be areas of need within the comprehensive model, as indicated by lower satisfaction within that group in communication from the respiratory therapist and education on sleep apnea, patients in this model have been shown to achieve higher levels of adoption, adherence, and persistence to sleep therapy vs. the traditional care paradigm. Future research should continue to guide the intersection of (1) what works best for patients, (2) what is affordable for payors, and (3) what achieves the adoption and adherence rates necessary to drive long-term health gains.

## Data Availability

The raw data supporting the conclusions of this article will be made available by the authors, without undue reservation.
